# Advances in the Prophylaxis of Respiratory Infections by the Nasal and the Oromucosal Route: Relevance to the Fight with the SARS-CoV-2 Pandemic

**DOI:** 10.3390/pharmaceutics14030530

**Published:** 2022-02-27

**Authors:** Nadezhda Ivanova, Yoana Sotirova, Georgi Gavrailov, Krastena Nikolova, Velichka Andonova

**Affiliations:** 1 Department of Pharmaceutical Technologies, Faculty of Pharmacy, Medical University of Varna, 55 Marin Drinov Str., 9000 Varna, Bulgaria; yoana.sotirova@mu-varna.bg (Y.S.); georgi.gavrailov@mu-varna.bg (G.G.); velichka.andonova@mu-varna.bg (V.A.); 2 Department of Physics and Biophysics, Faculty of Pharmacy, Medical University of Varna, 55 Marin Drinov Str., 9000 Varna, Bulgaria; krastena.nikolova@mu-varna.bg

**Keywords:** COVID-19, SARS-CoV-2, respiratory infections, prophylaxis, nasal drug delivery, mucosal drug delivery, protective nasal sprays, protective oral sprays, mucoadhesive dosage forms, antiviral drugs

## Abstract

In this time of COVID-19 pandemic, the strategies for prevention of the infection are a primary concern. Looking more globally on the subject and acknowledging the high degree of misuse of protective face masks from the population, we focused this review on alternative pharmaceutical developments eligible for self-defense against respiratory infections. In particular, the attention herein is directed to the nasal and oromucosal formulations intended to boost the local immunity, neutralize or mechanically “trap” the pathogens at the site of entry (nose or mouth). The current work presents a critical review of the contemporary methods of immune- and chemoprophylaxis and their suitability and applicability in topical mucosal dosage forms for SARS-CoV-2 prophylaxis.

## 1. Introduction

Respiratory tract infections (RTIs) are common and acquired predominantly via airborne and respiratory droplet transmission mechanisms [1,2]. One such infection—the SARS-CoV-2—continues to spread to the level of a pandemic [3] and provokes scientists to think critically, explore, question, and hypothesize in all aspects of the resultant ongoing crisis—from medical and pharmaceutical to social, psychological, economic, etc. At the same time, science today is advanced and progressive and has a vast resource to react flexibly and repurpose developments to suit the current urgencies and needs. The recent stepping into the market of new-generation mRNA vaccines for COVID-19 [4,5,6] is the biggest but far from the only proof of that. It is reasonable to assume that much of the reinforced research and development during the pandemic will also retain its value and find its contribution beyond the COVID-19 crisis. The current survey is focused on innovative developments for protection against RTIs, with an emphasis on nasal and oromucosal pharmaceutical formulations, eligible for self-use by the patients. The topic is of global and universal interest and most relevant for the aim of protection of individuals who encounter high risk for infection in their social environment—for example, by traveling, visiting a medical facility, and participating in public events.

The adhesion of a pathogenic microorganism to the site of entry in the human body is a determinative step for its further absorption, replication, and ability to cause disease [7]. The main entry sites for the most common bacteria and viruses are the mucosal surfaces of the nasal and oral cavity, the eyelids, and the rectal and vaginal tracts [7,8]. Although the aforementioned mucosal tissues differ in structure and function, they also have much in common. For instance, they all represent a far less effective barrier for pathogenic microorganisms than the skin and possess a morphology much more susceptible to the occurrence of microlesions. The latter are of great importance for the generalization of a locally arisen infection [8,9]. However, the mucosal tissue is covered by a mucus gel, which has a biophysical protective function on the underlying mucosal epithelium [10,11]. In terms of microbial contamination of the mucosal surface, the mucus and the mucociliary clearance play a key role in limiting the time of retention of the pathogen on the site of entry and its permeation to the lymph and blood flow [12].

Mucoadhesive polymers are widely used for their ability to attach to mucous surfaces and ensure prolonged contact of a drug-delivery system with the site of application [13]. If obtained with good spreadability, the mucoadhesive forms could provide a “shielding” film on the mucosa, enhancing its defensive properties against pathogens [14]. The use of mucoadhesive polymers in proper concentration for nasal and oromucosal administration is considered a novel and strategic approach for the aim of mucosal integrity restoration and prevention of respiratory infections [15,16,17]. Moreover, the introduction of active compounds with antimicrobial properties in mucoadhesive vehicles is highly relevant to the search for more efficient approaches in the prophylaxis of RTIs [14,15]. Since respired air is primarily routed through the nose, the nasal formulations are the ones of most importance [15].

## 2. An Insight of the Early Phases of Respiratory Tract Infections (RTIs)

The most common causes for primary RTIs are viruses [17,18] (*Influenza* viruses, *Coronaviruses*, *Rhinoviruses*, *Human metapneumovirus*, *Parainfluenza virus*, *Respiratory Syncytial Virus* (RSV), *Adenoviruses* [19,20]) and less frequently bacteria (*S. pneumoniae*, *H. influenzae*, *Moraxella catarrhalis* [21]). The *Coronaviruses* (including the lately identified SARS-CoV-2), and likewise the other pathogenic microorganisms in this group, transmit in the community predominantly via four mechanisms: (1) airborne—via small respiratory droplets, “droplet nuclei”, or dust (aerosol transmission), (2) respiratory droplet mechanism (via large respiratory droplets generated through coughing or sneezing), (3) direct contact (person-to-person transmission), and (4) indirect contact—transmission from an animate or inanimate (fomite) source [1,15,22,23]. Regardless of the route of transmittance, the upper respiratory tract is the most likely site of their entry into the human body [24].

The early stages of the viral and bacterial pathogenesis include adherence and implantation of the pathogenic microorganism at the portal of entry, viz., the respiratory mucosa [7,21,25,26]. The first-line, nonspecific natural opposing mechanisms to pathogen adherence are represented by the mucus secretion (covering the underlying tissue) and its motility, driven by the respiratory cilia (mucociliary clearance) [26], and the rapid replacement of mucosal epithelial cells (completed in about every 36–48 h) [21,25]. These forms of local resistance work in favor of hindered diffusion and shorter contact time of the pathogen with the living tissue. Some of the main prerequisites for unsuccessful timely pathogen clearance at this point are the existence of an accompanying infection [21] or chronic disease [26], compromised integrity of the mucosa (microlesions or wounds [21,25]), and smoking [26,27,28]. In addition, some viruses and bacteria express virulence factors that affect the mucociliary clearance efficacy and thus ease their own adherence [26]. Many pathogenic microbes, including *Streptococcus pneumoniae*, *Haemophilus influenzae*, *Coronavirus*, *Influenza*, RSV and *Rhinovirus*, are known to disrupt the ciliary beating and coordination [29,30,31,32,33,34,35,36,37].

**Figure 1:**
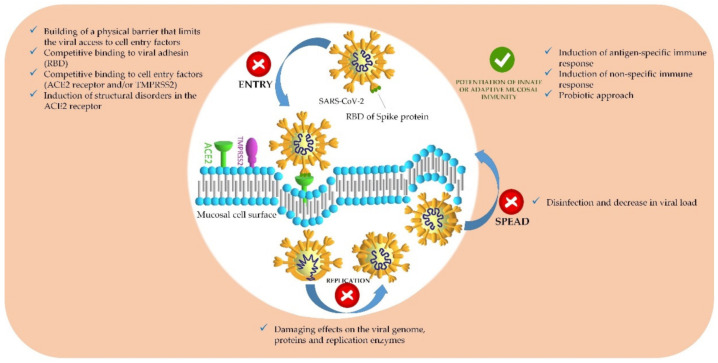
Infectious cycle of SARS-CoV-2 and niches for prevention of the infection.

If the mucus barrier is overcome, further implantation of the virus or bacteria is determined by the direct contact with the mucosal epithelia and bonding [7,21,25]. The attachment for most Gram-negative bacteria occurs via pili (fimbriae) [25]—thin, hair-like protein appendages on their surface that bind to specific membrane factors on the host cells [21,25,38]. Similarly, viruses use specific protein domains to establish a connection with certain receptors of the host cells; further on, they enter the cytoplasm (by endocytosis), access the cell’s synthetic apparatus, and use it for their own replication [7,39]. In general, the virulence factors expressed by the pathogen to serve for its attachment to the host cells are termed “adhesins” [40,41]. The role of a leading adhesin for all *Coronaviruses* is played by the surface glycoprotein Spike (S-protein) and its receptor-binding domain (RBD) (Figure 1) [15,39]. Examples of known targeted host receptors are the angiotensin-converting enzyme 2 (ACE2) for SARS-CoV and SARS-CoV-2 [42], dipeptidyl peptidase-4 (DPP4) for *Middle-East Respiratory Syndrome Coronavirus* (MERS-CoV) [43], human aminopeptidase N (CD13) for *Human Coronavirus* subtype HCoV-E229 [32], human leucocyte antigen class I (HLA class I) and sialic acid for *Human Coronavirus* subtype HCoV-OC43 [44,45,46], sialic acid for *Influenza* type A and B [46,47,48], intercellular adhesion molecule 1 (ICAM-1) for *Rhinovirus* [34,46,49,50], and CX3C chemokine receptor 1 (CX3CR 1) for RSV [36,37]. The genetically specified expression of these receptors on the cell membranes of differentiated cells dictates the cell/tissue tropism and spread mechanisms of the pathogen [39]. The ACE2 receptor for SARS-CoV-2, for instance, is abundant among the respiratory epithelial cells (with the ciliated bronchial epithelial cells and type II pneumocytes being highly enriched [42,51]), the oral and ocular mucosa, the salivary glands, and the intestinal enterocytes [52]. The stages of pathogen implantation and local replication are usually asymptomatic and related to the incubation period, peculiar for every infectious disease [7]. The onset of the symptomatic phase and the severity of the infection are determined by the pathogen’s virulence, infectivity, and initial dose exposure, as well as by the host resistance (immunity—specific and nonspecific; local or systemic) [7,25].

## 3. The Upper Respiratory Tract (URT)

The upper airway is presented by the nasal and oral cavity, the pharynx, and the larynx. Among its most important physiological functions is to deliver the inhaled air to the tracheal tree in an adjusted state—filtrated, warmed, and humidified [53]. The external respiration takes place through the nose or, occasionally, through the mouth. Although the air flows merge into the oropharynx toward the larynx, trachea, and lungs, it is important to distinguish the functional differences between the nasal and the oral passages, as they determine a different quality and safety of the breath [54].

### 3.1. The Nasal Cavity

**Figure 2:**
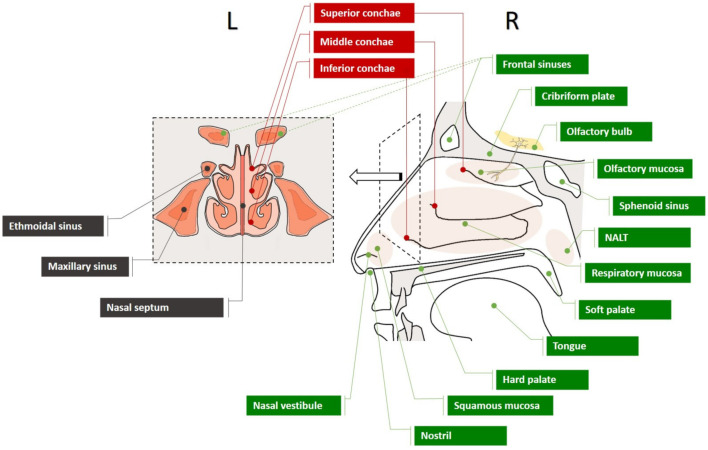
Structure of the nasal cavity: lateral (R) and frontal view (L).

The nasal architecture is composed of bony, cartilaginous, and soft matter units (skin/mucosa, muscles, fibroadipose tissue, vessels, nerves), building the external nose and the nasal cavity (Figure 2). Anteriorly to posteriorly, the nasal passage starts from the nostrils and the nasal vestibule (interior structures of the external nose) and opens to the nasal cavity. The nasal septum (“inner wall”) divides the nose and the nasal cavity medially into two symmetrical departments. From the bottom up, the nasal cavity expands from the hard palate (“floor”) to the cribriform plate (“roof”), whereas axially, three conchae (nasal turbinal) differentiate the inferior, middle, and superior zones (meatus). The total length of the nasal passage in adults is estimated at an average of 14 cm, and the total surface area at 160 cm^2^. Orifices on the lateral wall of the nasal cavity allow the drainage of the paranasal sinuses (frontal, ethmoid, maxillary, and sphenoid) and the connection to the Eustachian tube and nasolacrimal channel [55,56].

The external nose is covered by skin, which, in the zone of the nostrils and the nasal vestibule, is enriched in hair follicles, giving the growth of the nasal hair (vibrissae) [55]. The latter is responsible for the filtration of larger particles, such as pollen, bacteria, and dust [57]. The finer purification of the inspired air, however, is a function of the respiratory mucosa—the dominant outermost layer of the respiratory compartments, including the nasal cavity. It has a specific structure that allows the secretion and formation of the protective, covering nasal mucus and its motion toward the throat so that the potentially hazardous small particles in the breath, including those that are pathogen-containing, are swallowed instead of inhaled. This process is known as nasal mucociliary clearance and is a part of the overall mucociliary clearance of the respiratory system [26]. It is considered to effectively eliminate 95% of the particles larger than 15 µm in diameter [58]. However, a high percentage of the virus-containing droplets generated through talking, coughing, or sneezing reduce their size substantially due to evaporation (and become airborne) prior to their inhalation from the recipient [59,60]. Such particles are in the respiratory range and are less likely to be defeated by the nasal mucociliary clearance; thus, they may remain a threat to the immune system.

### 3.2. The Nasal Mucosa

The structural and functional features of the nasal mucosa are the key to understanding the physiological nasal defense mechanisms and lay the fundamentals of the biopharmaceutical concept for nasal drug delivery. A region of squamous epithelium follows the lining of haired skin in the nasal vestibule. Posteriorly, passing through a transitional zone, the tissue turns into respiratory mucosa, which covers most parts of the nasal cavity and, further, the pharynx, larynx, and lower airway [61]. The common structure of the mucous membranes includes an epithelium layer laid on a basement membrane that separates it from the underlying lamina propria—a connective tissue layer, host of vascular and neural plexuses, seromucous glands, and immune cells. The mucosa is supported by a thin muscle layer (muscularis mucosa), after which the submucosal tissue (tela submucosa) begins—a connective tissue that nourishes (serves) the mucosa (also contains glands, nerves, and blood vessels). The nasal respiratory epithelium is distinguished by ciliated cells distributed among an assembly of columnar and goblet cells. The former cell type—ciliated cells—is characterized by an elongated (columnar) body, an apical surface of microvilli (cytoplasmic, fingerlike formations), and up to 300 thin, tubular, and mobile appendices (cilia) striking out up to 5 µm of the microvilli bed. The microvilli-type morphology (also intrinsic for the apical surface of nonciliated columnar cells) significantly increases the total surface area of the mucus membrane and prevents it from dehydration, while the cilia ensure the motion of the mucus [55,56]. The coordinated movement of the cilia is termed “ciliary beating”—a result of synchronized alternation of effective and recovery ciliary strokes triggered by the ATP-dependent motor protein dynein [62]. The goblet cells execute a secretory function, wherefore they are also referred to as unicellular glands. Their nuclei, being displaced toward the cell base, open space for the storage of mucin-containing granules at the apical side, where their release takes place and contributes to the lubrication of the mucosal surface. The density of goblet cells in the epithelial layer significantly increases during inflammation [55]. In addition to the squamous, transitional, and respiratory mucosa, two other zones are also distinguished in the nasal cavity—the olfactory and the lymphoid regions. The olfactory mucosa differentiates from the respiratory mucosa by several parameters: (1) it contains several other cell types specified to provide its chemoreceptive function (olfactory, supporting, and brush cells); (2) the density of ciliated cells is much lower; (3) the olfactory epithelium does not include goblet cells; (4) the serous glands situated in the lamina propria layer are called Bowman’s glands and produce less viscous fluid (compared to the respiratory mucus), which makes it more suitable for cleaning (washing) the sensory zones and dissolving substrates [55,56]. The lymphoepithelium (NALT—nasopharynx-associated lymphoid tissue) is the zone with the most significance to the immune response in the nasal cavity, for, in its lamina propria layer, clusters of antigen-responding M cells are found [63,64]. In adults, it is strategically located at the pharyngeal entrance and presented in the structure of the tonsils in the Waldeyer’s ring (the palatine, tubal, adenoid, and lingual tonsils) [65].

The nasal cavity is abundantly vascularized, which allows the effective processing of the inspired air to optimal humidity and temperature. The predominant blood supply is ensured by the sphenopalatine artery and the ethmoidal branches of the ophthalmic artery. Blood drainage occurs through the ethmoidal veins to the pterygoid, cavernous, and ophthalmic plexuses. The general innervation of the nasal mucosa derives from branches of the maxillary and ophthalmic nerves, whereas the sensory innervation, relating to the sense of smell, is a function of the olfactory nerves [55,58]. The latter enter the nasal cavity through openings in the cribriform plate, innervate the olfactory region (located in the uppermost part of the cavity), and connect to the olfactory bulb in the cranial cavity [66]. Thus, they directly relate the nasal cavity with the brain.

### 3.3. The Mucus

The nasal mucus is a 10–15 µm thick, fluid biophysical barrier covering the nasal mucosa and protecting it from potentially hazardous agents having reached the intraluminal space (substances and pathogens) [58]. It serves as a mobile carrier for entrapped pathogens, particulate matter, and dissolved substrates and is moved by the ciliary beating at an average velocity of 2–25 mm per minute [67]. At the same time, it hosts and mediates the defense action of immunoglobulins (IgA and IgG) [68,69], immune cells (neutrophils, monocytes, macrophages), nitric oxide (NO), enzymes (lysozyme), proteins (lactoferrin), and the protective microflora—all produced and/or present in the nasal mucosa and responsible for its specific or nonspecific immune response [58]. The liquid milieu of the mucus is provided predominantly by the exocrine seromucous glands and, to a lesser extent, by the plasma transudation and the tear fluid [58,61]. However, the viscoelastic structure of the nasal mucus is defined by the presence of mucins (MUC)—glycoproteins, synthesized and secreted by the goblet cells [55]. Several types of mucins are identified in the nasal fluid—MUC5AC and MUC5B, which are cross-linked and gel-forming mucins, and MUC1 and MUC4, which are cell-surface mucins. Their distinct distribution determines a two-phase state of the nasal mucus. The first, lower phase (pericilliary layer) is characterized by low viscosity and lubricates the microvilli. It contains the cell-surface mucins, which are dominant glycocalyx constituents, and separates the pericilliary layer from the upper-laying viscous fluid. The latter is considered a second phase of the nasal mucus, in which the oligomerizing and network-forming MUC5AC and MUC5B design a gel-like structure [8,26]. The mucins possess a highly reactive structure, which can bind to various substrates, including microbial adhesins, drugs, and macromolecules, via hydrogen bonding, disulfide bridges, Van der Waals attraction, or other mechanisms. The presence of sialic acids and sulfated groups in the saccharide parts of the mucins provides a negative charge and slightly acid physiological pH (5.5–6.5) of the nasal mucus [58]. The volume and viscosity of the mucus are regulated by macro- and microenvironmental factors and influenced by the presence of inflammatory processes, microbial infections, irritants, and other [55].

### 3.4. The Role of the Oral Cavity as a Portal of Entry for Respiratory Pathogens

The oral cavity may become an entry point for respiratory pathogens through the oral–lung axis, oromucosal infection and/or absorption, or even the oral–gut axis [70,71,72,73]. The former mechanism relates to the act of oral inhalation (mouth breathing) or microaspiration, by which a respiratory pathogen bypasses the antigen-resilient nasopharyngeal zone and reaches the lower airways [74,75]. For most people, occasional mouth breathing (partial—combined or alternating with nasal breathing, or absolute) is inevitable during physical exercise, sleeping, singing, talking, etc. [76]. In contrast, habitual mouth breathing (due to obstructive nasal pathologies, such as allergic rhinitis, recurrent infections, and deviated septum [77]) is defined as an abnormal respiratory pattern [78]. Indeed, for healthy individuals, wearing a face mask is one prerequisite for sporadic mouth breathing, resulting from the intention to compensate the airflow. The oral inspiration is considered riskier for respiratory infections since the breath is devoid of several defense mechanisms inherent for the nasal air processing; these include control over the airflow, filtration, humidification, and antimicrobial processing through reactive species produced in the nasal cavity (e.g., nitric oxide—NO) [79]. The antimicrobial activity of NO has been highlighted by many scientists recently, confirming the importance of nasal breathing for the host’s natural defense capacity against SARS-CoV-2 infection [80,81,82].

Oral pathogen entry could also be observed via oromucosal or gastro-intestinal infection and absorption. The oral cavity possesses, adjusted to its physiological designation, high enzymatic activity, diverse microbiota, mechanically sustainable stratified mucosal epithelium, salivary clearance, and a mucosal-associated lymphoid tissue region (MALT) [83,84]. Yet, these barriers are not absolute and, under certain circumstances (impaired integrity of the mucosa, nutritional and other habits, accompanying disease, compromised microflora, etc.), the oral mucosa can serve as a site of adherence and absorption for respiratory pathogens [85]. One eloquent example could be given again for the novel *Coronavirus*, which is currently reported to adhere to and infect salivary glands cells and oromucosal epithelial cells, especially in the tongue and gingival area (a mechanism related to the commonly observed loss-of-taste symptom of COVID-19 infection). This fact is explained by the high density of the SARS-CoV-2 entry factors in the oral tissues in question, i.e., the ACE2 receptor and the transmembrane serine proteases 2 (TMPRSS2) [70,71,86,87,88]. Furthermore, although a respiratory virus by definition, the SARS-CoV-2 is described to infect the intestinal enterocytes (also expressing the coronaviral cell entry factors) through the oral–gut axis and systemically spread through the gut–lung axis [89,90,91,92]. The risk of such a form of infection is increased under the conditions of lowered stomach acidity (above pH 3, where the virus survival rate is higher) [93,94]. Similar findings are reported for MERS-CoV [95]. In addition to the risk of primary infection through the oral structures, oral hygiene and immunity are crucial for the prevention of co-infection, which can arise in the same aforementioned manners [75,96].

## 4. Prevention and Prophylaxis of RTI’s

The prevention of infectious diseases is a priority mission of the healthcare system, insured by a complex algorithm of actions focused on limiting the spread and morbidity. The public health measures include recommendations for effective personal hygiene, the imposition of behavioral norms in a social environment (the adherence to a social distance, the wear of a mask, the abstinence from social contacts in case of symptomatic discomfort, quarantine, etc.), regular screening and testing, and other [97,98,99]. Although all individuals benefit from an effectively maintained epidemiological situation, most often, they are not the subject of protection; the community is. How successful the preventive measures of this kind will be for an individual depends on many factors, including their own understanding and compliance. In the light of the ongoing COVID-19 pandemic, the misuse of protective supplies, such as face masks, is one eloquent example for the above. Many research studies emphasize the necessity of correct and consistent application of the face-covering masks in order for them to significantly decrease the risk of infection [100,101]. However, the proper implementation of this measure has been long proven to suffer from a low compliance among the population [102]. Furthermore, particular materials possess a reduced capacity to effectively filtrate bacteria and virus-containing particles from inspired/expired air [103,104,105,106]. Last but not least, circumstantial or associated with the incorrect use, insufficient efficacy of masks and even increased risk of infection are being reported [107,108,109,110,111,112].

Prophylaxis is an integral part of prevention with much higher significance to personal health. It consists of actions taken to provide protective treatment for a specific disease or a group of diseases and is often strictly related to the pathogenesis [113]. In the context of respiratory infections, pre- and post-exposure to pathogen prophylactic measures are to be distinguished. In fact, some of the methods of prophylaxis are effective in both phases. Prophylactic treatment is carried out after a physician’s prescription or recommendation or as a result of patients’ self-awareness when it concerns the use of well-known and over-the-counter products. Two major branches of the prophylactic treatment of respiratory infections are immunoprophylaxis and chemoprophylaxis. The former method refers to the administration of vaccines, serums, or immunoglobulins, whereas the latter describes the application of chemotherapeutic agents for the aim of prevention. The most significant and relevant prophylaxis treatment methods (approved or in a research phase) for RTIs will be discussed.

### 4.1. Immunoprophylaxis

Immunoprophylaxis is, indisputably, the most effective preventive step to be taken in the name of limiting the morbidity and mortality due to RTIs. Vaccination, also known as active immunization, is an available option against *S. pneumoniae*, *H. influenzae* (type b), and *Influenza*, and recently, against the novel *Coronavirus*—SARS-CoV-2. Several technologies are recognized in vaccine development. They include the use of the whole pathogen (live-attenuated and inactivated vaccines), viral and bacterial vectors, nucleic acid antigens (mRNA and DNA vaccines), subunits (e.g., purified protein or recombinant protein vaccines), polysaccharide–protein conjugates, toxoids, virus-like particles, outer membrane vesicles, and antigen-presenting cells [114]. The main setbacks of vaccination are related to the pathogen variability, short-term immune response, or low efficacy [115]. Furthermore, it is not the “first choice” preventive measure for immunocompromised or chronically ill patients [116]. The mucosal vaccination, in this regard, offers some advantages, as it allows antigen-specific local, as well as systemic, immune response stimulation through a nonparenteral route of administration (such as intranasal and oral) [117]. Passive immunization, on the other hand, is an alternative to vaccination by administration of antibody preparations (polyclonal antibodies-containing serums or monoclonal antibodies) [118,119]. The passive immunoprophylaxis is characterized by a much lower risk of side effects, compared to the vaccine administration, and faster onset of activity, which is highly valuable for hospitalized patients in a post-exposure stage [115,119,120]; however, a passively acquired immunity lasts for a shorter time and requires a continuous supply of the immune preparation [115,119]. An interesting branch of passive immunoprophylaxis, with practical significance in the prevention of RTIs, is the administration of immunoglobulin Y (IgY). The is derived from the egg yolks of birds immunized with specific antigens and represents a functional analog of the mammalian immunoglobulin G (IgG) [121]. IgY immunotherapy distinguishes with numerous advantages over conventional antibody preparations—high tolerability and safety [115,119], longer circulating half-life and greater potential for antigen binding (compared to IgG) [122], ecologically and animal-friendly technology of production [115,123], and lower production costs (compared to those for monoclonal antibodies) [119]. So far, the efficacy of IgY immunotherapy has been proven for the prevention of *Influenza* [115,119], *Pseudomonas aeruginosa* [124], *Mycobacterium tuberculosis* [125], bovine RSV [126], SARS-CoV [127], and SARS-CoV-2 [128] infections, and other pulmonary diseases.

### 4.2. Chemoprophylaxis

The term chemoprophylaxis relates to the preventive administration of antimicrobial agents in the pre- or post-exposure stage [129,130]. Key targets for the drug action are the pathogens themselves (inhibition of adhesion, entry, replication) or the host cell factors [131].

Long established in clinical practice is the prophylactic use of antibiotics and, less frequently, of antiviral agents [132,133]. Despite the great health benefits of prophylactic antibiotic treatment, especially among hospitalized and immunocompromised patients [134,135,136], it remains a leading cause of the major and global problem of antibiotic resistance [137,138]. The administration of some antiviral drug representatives with neuraminidase inhibitory mechanism of action (*Oseltamivir*, *Zanamivir*) is approved for the prophylactic treatment of *Influenza* [116]. However, their use is more relevant for patients at high risk than for healthy adults due to the substantial side effects and the often unfavorable risk–benefit ratio [139]. At present, therapy with *Bamlanivimab* 700 mg + *Etesevimab* 1400 mg (intravenous infusion) or *Casirivimab* 600 mg + *Imdevimab* 600 mg (subcutaneous injections or intravenous infusion) is the current World Health Organization’s recommendation for post-exposure prophylaxis of *Coronavirus* (SARS-CoV-2). In contrast, no drug has been officially recommended for pre-exposure prophylaxis [140]. Under investigation in clinical trials are the efficacy and safety of several agents in pre- or post-exposure stage, including *Tenofovir* and *Emtricitabine* (separately or in combination), *Hydroxychloroquine*, *Ivermectin*, and interferons [140,141]. Furthermore, the benefits of supplements such as zinc, vitamin C, and vitamin D are also under continuous research, despite their long-standing use and known efficacy in prophylaxis of other RTIs [140].

Active substances or preparations of natural origin (herbal, animal, fungal, bacterial, yeast) are widely studied and used for prophylaxis of RTIs. Their application in medicinal products is preferred and prioritized due to the higher safety profile and increased patient compliance. The antimicrobial (and especially antiviral) activity of natural components is a trendy subject of research and is leading to the accumulation of more and more scientifically based facts to support the pharmaceutical development of natural products for RTI prophylaxis [142,143,144]. Unlike most synthetic drugs, bioactive compounds (BACs) often possess a complex mechanism of action, determining a broad spectrum of activity and efficacy in different stages of the infectious disease [141,145]. Some biomolecules (primary or secondary metabolites), including polysaccharides, glycoproteins, flavonoids, and other phenolic compounds, alkaloids, and terpenoids, are recognized for their ability to competitively bind to the host cell receptors or the viral adhesins in an antibody-like pattern and prevent the receptor-determined cell entry of the pathogenic microorganisms [14,142,143,144]. This fact is to be explained with a structural similarity of many BACs with segments of the targeted host receptors and adhesins [142] and/or high reactivity (e.g., high hydrogen bond donor and acceptor number [144], chelating ability). More than 90 natural compounds of the above-mentioned phytochemical categories have shown anti-coronavirus activity [142]. The latest studies and reviews focus the attention on S-containing polysaccharides (*Chitosan* [146] and semisynthetic derivatives [147], kappa-, iota-, lambda-*Carrageenan* [148,149,150], *Fucoidan* [150,151,152]), *Lactoferrin* [153,154], *Aloe vera* polysaccharides [155], the flavonoids *Hesperidin* [14,156,157], *Hispidulin* [158], *Quercetin* [153], *Rutin* [159], *Resveratrol* [160], the saponin *Glycyrrhizin* [161], and the diterpenoid *Andrographolide* [162,163]. Many of them possess not only the ability to block the adhesin–receptor connection and following viral entry, but are also proven to exhibit antiviral activity due to inhibition of viral replication (*Chitosan* and other sulfated polysaccharides [145,164,165], *Resveratrol* [166,167], *Aloe vera* polysaccharides [155], *Glycyrrhizin* [168], *Andrographolide* [169]), inhibition of protein synthesis (*Aloe vera* polysaccharides [155] and *Andrographolide* [169]), blocking the viral release and spread (*Andrographolide* [169]), and/or immunomodulatory properties (*Chitosan* [170]). The antiviral potency of other biomolecules is primarily due to interference in one or multiple steps of the infectious cycle of pathogens (i.e., after adhesion) and by activating the host immunomodulatory system. Examples are *Echinacea* spp. phenolic glycosides and alkamides (immunomodulatory effects on macrophages and NK cells [144,171]), *Astragalus* spp. polysaccharides (inhibition of viral replication, and immunomodulatory effects on macrophages and NK cells [172,173]), *Rhodiola rosea* flavonoids (inhibition of viral replication and immunity enhancement [174]), and alginate (immunity enhancement [175]). Not by accident, some of the polysaccharides above (*Chitosan*, *Astragalus* spp. polysaccharides, *Carrageenan*, and *Alginate*) are proposed by many authors as potent vaccine adjuvants to improve immune response [170,176,177,178,179,180].

Probiotic supplementation is another strategy based on natural preparations worth mentioning in RTI prophylaxis. The beneficial effect of probiotic strains on the duration and severity of RTIs, including *Rhinovirus*, *Influenza*, RSV, and *S. pneumoniae* infections, has been long studied and established [181,182,183]. It is owed to several mechanisms, among which the most important are (1) immunomodulation—probiotic bacteria comprising immunostimulatory constituents such as peptidoglycan, lipoteichoic acid, Toll-like receptor ligands, and muramyl dipeptide; their use leads to an increase in the level of type I interferons, the number and activity of natural killer (NK) cells, T cells, and IgA-expressing B cells in the colon and lymph nodes, and the level of specific antibodies in the lungs [184,185]; (2) production of antimicrobial substances (lactic acid, hydrogen peroxide, and bacteriocins) [185]; (3) competitive adhesion of probiotic bacteria on the sites of potential colonization of pathogenic bacteria [186], and (4) interaction with receptors, binding domains, and suppression of toxin-mediated responses [186]. An interesting finding of Verma et al. on *Lactobacillus paracasei* strains suggests a potential anti-SARS-CoV-2 activity [187]. The team reveals the expression of human ACE2 in the probiotic bacteria in question, which could prevent the viral cell entry by interaction with the S-protein of the virus [185,187].

As the implementation of nanotechnologies is an integral part of contemporary and innovative science, many nanotechnology-based attempts are being made to improve the pharmacological and biopharmaceutical properties of effective active ingredients in RTI prophylaxis. Nanomaterials find their application in the production of safety supplies (e.g., nanofibrous materials for the production of protective clothing, face masks and filters, enriched or not with antimicrobial agents [188,189,190]), in the composition of targeted and sustained drug delivery forms (e.g., cellular nanosponges from plasma membranes of human lung epithelial type II cells or human macrophages, containing protein receptors to bind and neutralize SARS-CoV-2 [191]; poly(lactide-co-glycolide)-b-poly-(ethylene glycol)-maleimide polymeric *Ivermectin*-loaded nanoparticles for sustained drug delivery [192]), and in gene delivery (e.g., lipid nanoparticles as vehicles for targeted intracellular delivery of mRNA [193,194]—a technology used in some SARS-CoV-2 vaccines).

The mechanisms of action of the various therapeutics—synthetic or natural, included or not in nano-carriers—suggest that they could be potentially effective in RTI prophylaxis if not only systemically but also nasally or oromucosally applied. The anatomo-physiological peculiarities of the upper respiratory tract and the biopharmaceutical characteristics of the nasal and oromucosal route for drug delivery are in tight relationship with the choice of a dosage form and active ingredient and the possible beneficial result for the local protection against respiratory infections.

## 5. The Nasal and Oromucosal Drug Delivery

Nasal and oromucosal drug delivery is a contemporary field of research, mostly because of the multiple advantages they offer beyond their most commercial designation—to treat and/or protect the mucosa. The nasal route stands out as one of the most prospective sites for peptide and protein delivery [195], and direct brain drug delivery (via the olfactory bulb) [196,197]; the buccal route reveals opportunities for sustained drug delivery, and the sublingual route allows fast access to the systemic circulation [198,199]; both the nasal and the oromucosal routes are eligible for mucosal vaccination, for which target zones are the lymphoid regions (NALT and MALT, respectively) [65,200,201,202,203]. However, in the light of the current topic, the focus will be directed to nasal and oromucosal drug administration for local protection.

A protective mucosal formulation might be designed to strengthen the biophysical barrier properties of the mucus and be suitable for reconstructive aims, exert a local pharmacological activity, and/or disinfect. In general, the formation of a stable, even, and retentive film on the mucus membrane is required, with the capacity to ensure an adequate drug diffusion to the target structures (if the formulation is drug-loaded) while not interfering with the physiological functions at the site of application (e.g., inhalation, mucociliary clearance, smell, chewing, swallowing, and talking). Exceptions are the rinsing solutions and the mucosal vaccines, as the former are not intended to adhere, whereas the latter are intended to fully cover the mucosal surface (they are designated to be delivered to specific zones in the cavities).

### 5.1. Mucoadhesion—Principles and Utilization in Mucosal Dosage Forms

Mucoadhesion is the phenomenon defined by the sustainable adhesive attraction between a mucosal surface and a second biological or synthetic (most often polymeric) substrate. The utilization of mucoadhesive excipients is an integral part of the design of mucosal dosage forms since they ensure a prolonged retention, intimate contact with the mucosa, and resilience of the emerging film to gravity and unfavorable physiological processes at the site of application (such as the nasal mucociliary clearance and the great oscillatory stress in the oral cavity) [13,204]. Mucoadhesive properties are inherent for many polymeric materials of natural and semisynthetic origin that possess the ability to interact with the mucus mucins, the mucus milieu, and/or the mucosal epithelia in their hydrated state. The class of mucoadhesive polymers is heterogeneous and diverse; it includes polysaccharides (e.g., natural gums—*Xanthan*, *Guar*, *Carob*; *Chitosan*, *Carrageenan*, *Sodium Alginate*, *Hyaluronic Acid*, and derivatives), acrylic/ methacrylic polymers (e.g., *Carbomer* and *Polycarbophil*), cellulose derivatives (*Hydroxypropylmethyl cellulose*, *Hydroxyethyl cellulose*, *Hydroxypropyl cellulose*, *Carboxymethyl cellulose* (sodium)), and many other (e.g., *Poly(Ethylene Oxide)*, *Poly(Vinyl) Alcohol*, *Poly(Vinyl) Pyrrolidone*) [205]. A mucoadhesive connection may be owed to physical phenomena (e.g., hydrogen bonds, van der Waals forces, hydrophobic interactions), physico-chemical events (e.g., wetting, swelling, and dissolution of the mucoadhesive polymers, diffusion, and adsorption of the polymer chains into the mucus matrix and intercellular space of the mucus membrane), and/or specific or nonspecific chemical interactions (e.g., ionic or covalent bonds, including disulfide bonds and intermolecular cross-linking) [205,206]. The nature of the occurring interactions is determinative for the strength of adhesion. For example, thiomers (thiolated polymers, e.g., thiolated *Chitosan, Alginate, Poly (Acrylic Acid)*) are among the most potent mucoadhesives due to their capability to covalently bind to the cysteine-rich mucins through disulfide bridges [207,208,209]. Other important structural peculiarities of a mucoadhesive polymer are the presence and prevalence of amino, hydroxyl, and carboxyl groups, which get engaged in hydrogen bonding with mucins and cell-surface molecules [209,210,211]; the degree of intramolecular cross-linking and hydrophilic–lipophilic properties, which should be balanced (or optimized) with a view of sufficient molecular flexibility, but satisfactory sustainability of the applied protective/drug-delivery layer at the same time; the charge density, as it correlates to stronger adhesion (anionic and cationic polymers bind more efficiently than neutral ones do); the molecular mass, as usually, longer polymer chains contribute to better penetration and contact with the mucus and the underlying mucosal epithelium. The concentration of the mucoadhesive polymer in the pharmaceutical formulation, the natural lubrication of the mucosa, and pH also affect the mucoadhesion efficacy [205,206].

### 5.2. Nasal Dosage Forms

The nasal dosage forms include drops and liquid sprays, powders, rinses, semisolid preparations (gels, creams, or ointments), and sticks [212]. With the exception of the latter two groups, all the other forms are eligible for the aims of local protection of the nasal mucosa.

The nasal drops have the advantage of being well-tolerated and not “aggressive” to the mucosa, but upon the standard recommendations for administration, they reach and deposit on the posterior nasal cavity. Because of the higher cilia density and the greater permeability of the posterior mucosal site, they stimulate fast mucociliary clearance and drug absorption and are only preferred when a quick onset of pharmacological action is needed. Contrariwise, the nasal sprays ensure a distribution at the anterior nose and further spread and coverage of the posterior parts due to the mucociliary clearance [213,214]. Therefore, they are recognized as the better choice for delivering and retaining nasal liquids for protective purposes. However, the atomization pattern, and thus the type of nozzle and pump in the spray device, is of great importance for the spreading and localization of the applied dose [215,216].

The nasal powders offer an alternative to liquid forms with greater physicochemical and microbiological stability, prolonged retention, and drug absorption. Furthermore, they allow the administration of larger doses and do not set a requirement for solubility of the drugs and excipients. However, these solid nasal forms are still not widely introduced to the pharmaceutical market due to the higher production costs, lower mucosal tolerability, and patient compliance [217].

The nasal rinses are intended to be administrated in larger volumes, and wash and clear the nasal cavity from potentially hazardous agents. Most often, they are isotonic solutions enriched or not with antiseptics [212].

Regardless of the type of dosage form, the most significant challenges to the protective nasal formulations are the fast mucociliary clearance and the potential ciliotoxicity of particular constituents [212]. Although mucoadhesive polymers and viscosity enhancers are known to oppose the natural clearance mechanisms in the nasal cavity, quite often, the principles of their action contradict the provision of other desired qualities. The sustainability (mechanical strength) of a mucosally applied film, for example, increases in the presence of poor water-soluble polymers (with greater hydrophobicity or high degree of cross-linking), whereas such polymers indeed lower the mucoadhesive capacity [204]. The higher concentration of mucoadhesive polymers and the greater viscosity and yield stress of the formulation normally favor endurable retention and stability of the mucoadhesive layer but violate sprayability and spreadability [218,219]. The elaboration of in situ gelling systems, in this regard, is an irreplaceable tool. The systems in question ensure an adopted temperature-, ion-, or pH-dependent sol-gel transition of the formulation after application [220,221]. Commonly used polymers in nasal forms for that purpose are the Poloxamers (poly(ethylene oxide)/poly(propylene oxide) copolymers), Gellan gum, Carbomer, and others [220,221,222,223]. As so many requirements are to be met, the protective nasal dosage forms formulation often relies on the combination of different polymers, each contributing to or balancing certain features [15]. Within the choice of excipients, it is not to be forgotten that some polymers, especially those of natural origin, exhibit antiviral and antibacterial properties themselves [224]; thus, they would be highly preferable multifunctional constituents of a protective nasal formulation.

Ciliotoxicity is defined as the ability of particular substances to disrupt the regular ciliary beating frequency and, therefore, negatively affect the natural nasal ciliary clearance. Cilitoxic properties are known for most preservatives, “xerogel”-forming cellulose polymers, local anesthetics, corticosteroids, and antibiotics. The effects may vary from temporary and reversible to irreversible and inhibitory. In order to diminish the possible ciliotoxicity of a nasal preparation, the use of buffers (pH 6–8), tonicity-adjusting agents, and humectants is recommended [212,225].

The drug absorption pathways in the nasal cavity are not a primary concern when it comes to the development of a locally active formulation. However, for drug-containing formulas, they still need to be taken into account in order to predict or prevent an undesired systemic intake. Because of the rich blood supply and lymph drainage in the nasal area, therapeutic molecules responding to certain physico-chemical criteria may reach the systemic blood circulation and induce a systemic response. The olfactory region is a particularly critical zone, where a molecular or even particulate uptake may occur through the olfactory nerves to the brain; such uptake is even possible for pathogenic microorganisms [226,227,228]. Therefore, the risk of microbial contamination of nasal pharmaceutical formulations should be addressed and eliminated.

### 5.3. Oromucosal Dosage Forms

The oromucosal dosage forms are diverse preparations intended to be applied in the oral cavity and exert local or systemic effects. Convenient for protective purposes are easily spreadable forms, such as oral rinse solutions, gargles, sprays, lozenges, and pastilles [17,229,230,231,232]. Most commonly, they target the oropharyngeal and/or the buccal mucosa and contain wide-spectrum antiseptics and antimicrobial agents designated to disinfect the oral cavity. Undesired systemic drug intake across the highly vascularized buccal mucosa is possible for drugs with good permeability [199].

Unexceptionally, the oromucosal forms may contain mucoadhesive polymers to retain and build a protective barrier against irritants and pathogens or cover infection-susceptible lesions. However, the typically high levels of oscillatory stress in the oral cavity throughout the daily routine of chewing, drinking, swallowing, talking, etc., and the significant salivary flow (at approximately 1–2 mL/min [233]), limit the time of retention and determine the necessity of frequent application. As an alternative, buccal gel-forming tablets and buccal films, although predominantly used as modified-release drug delivery systems for systemic absorption, could also be applied for local protection [234,235].

Table 1 summarizes data from the latest studies on nasally and/or orally active therapeutics in RTI prophylaxis, relevant to SARS-CoV-2 infection prevention. The list includes new and repurposed drugs, formulations, and marketed products, scientifically proven for their efficacy against SARS-CoV-2.

Beyond the active agents listed in Table 1, many more candidates are hypothesized but not yet studied, in either in vitro or in vivo experiments, for their actual efficacy in the nasal and oral prophylaxis of SARS-CoV-2. Such are phenolic compounds with proven efficacy against other respiratory viruses (e.g., *Quercetin*, *Hesperidin*, *Diosmin*, *Resveratrol*) [167,305,306,307,308], essential oils [309,310,311,312], quaternary ammonium compounds with broad-spectrum antimicrobial activity [313] (e.g., *Cetylpyridinium chloride*, available in the nasal spray product “Halo™”(Oasis Consumer Healthcare, USA) [17]), statins [314], and others strictly related to the SARS-CoV-2 entry mechanism therapeutics (e.g., ACE2 agonists [315] and ACE2-coated nanoparticles [316]). On the other hand, some very potent antiseptics—viz., *Hydrogen peroxide* and *Chlorhexidine*, have not demonstrated the expected efficacy against SARS-CoV-2 [317,318,319]. Thus, to avoid speculation, thorough investigations are required to prove the applicability of each potential candidate in the local prophylaxis of the currently most significant threat for respiratory infection—the SARS-CoV-2 virus.

## 6. Conclusions

The nasal and oral cavities play an essential and determinative role for the development of a respiratory infection since they establish the first contact with the airborne and respiratory droplet-transmitted pathogens. It is, therefore, a primary goal to focus on the mucosal immunity and defense capacity, especially in the time of COVID-19 pandemic. As our research shows, more than 30 well-known or expressly modified molecules in the past two years are being investigated for their potency to improve the local resistance of the nasal and oral pathways against SARS-CoV-2. Although many are showing promising results, the delivery form/vehicle and the delivery device are addressed and are a subject of optimization in very few of these studies. It is our opinion that a thorough biopharmaceutical approach may contribute to extending the anti-SARS-CoV-2 capacity of even more therapeutics. Furthermore, regarding the nasal and oromucosal protective formulations, two other niches exist that have not been so extensively explored in terms of COVID-19 infection—the ability of such forms to restore the sense of smell and taste and reduce viral spread.

## Figures and Tables

**Table 1:** List of nasally and/or oromucosally administrated therapeutics for SARS-CoV-2 prophylaxis.

Active Agent	Form of Application	Type of Studies Conducted/in Progress	Efficacy	Mechanism of Action	Available Marketed Product(s)	References
**I. ANTIGEN-SPECIFIC PROPHYLAXIS**						
Adenovirus-vectored vaccine encoding the spike (S)-protein/RBD domain of SARS-CoV-2	Intranasal	In vivo preclinical Clinical trials	SARS-CoV-2	Induction of S-specific and receptor binding domain (RBD)-specific serum and secretory antibodies (IgG and IgA, respectively), and lung-resident T cells	n/a ^a^	[236,237,238,239,240,241,242]
Genetically modified live-attenuated vaccines	Intranasal	In vivo preclinical Clinical trial	SARS-CoV-2	Induction of S-specific and receptor binding domain (RBD)-specific secretory antibodies (IgA)	n/a	[243,244,245]
S-protein embedded bacterial outer membrane vesicles	Intranasal	In vivo preclinical	SARS-CoV-2	Induction of SARS-CoV-2-specific neutralizing antibodies	n/a	[246]
Monoclonal IgM antibodies	Intranasal	In vivo preclinical	SARS-CoV-2	Antigen-specific binding to SARS-CoV-2 RBD domain	n/a	[247,248]
Monoclonal IgG antibodies	Nasal spray	In vivo preclinical	SARS-CoV-2	Antigen-specific binding to SARS-CoV-2 RBD domain	InvisiMask™ (Eureka Therapeutics, USA)	[249,250]
**II. BROAD-SPECTRUM ANTISEPTICS**						
*Povidone-iodine* (PVP-I)	Oral rinse	In vitro Clinical trials	Broad-spectrum antimicrobial activity, including against SARS-CoV, MERS, and SARS-CoV-2	Oxidation-determined damaging on microbial nucleic acids, proteins, and cell membranes	Betadine^®^ 1% (Avrio Health L.P. Purdue Pharma Inc., USA), Halodine^®^ 1.7% (Halodine LLC, USA)	[230,251,252,253,254,255,256,257,258,259,260]
	Gargling solution				Betadine^®^ gargle 0.5% (Avrio Health L.P. Pur-due Pharma Inc., USA)	
	Oral and nasal spray	Clinical trials			n/a	
	Nasal irrigation	Clinical trial			n/a	
*Chlorine dioxide* (stabilized)	Oral rinse	In vitro	Broad-spectrum antimicrobial activity, including against HCoV-229E, *Influenza* A, SARS-CoV, and SARS-CoV-2	Oxidation-determined damaging on microbial nucleic acids, proteins, and cell membranes	ClōSYS^®^ oral care products (Rowpar Pharmaceuticals, Inc., USA)	[231,261]
*Hypochlorous acid* (HCLO)	Nasal and oral spray	In vitro Clinical trial	Broad-spectrum antibacterial activity, including against SARS-CoV-2	Oxidation-determined damaging on viral genome	n/a Tehclo™ technology platform (APR Nanotechnologies SA, Switzerland) for delivery of HCLO—Acid-Oxidizing Solution (AOS2020) is used	[262,263]
**III. POLYMERIC ANTIMICROBIAL AGENTS**						
*Hydroxypropyl methyl cellulose*	Nasal spray, Nasal powder spray	In vitro In vivo, on human volunteers	Nonspecific mucosal protection against respiratory-transmitted pathogens	Formation of a physical barrier on mucosa to facilitate the process of pathogen entrapment and clearance; additionally, Taffix™ (Nasus Pharma Ltd., Israel) nasal spray creates an acidic microenviroment, unfavorable for most respiratory pathogens	Taffix™ (Nasus Pharma Ltd., Israel) nasal spray, Nasaleze nasal spray	[264,265]
*Astodrimer sodium*	Nasal spray	In vitro In vivo preclinical	Broad-spectrum antiviral activity, including against SARS-CoV-2	Blocking of viral adhesion and entry by binding to viral adhesins and cell entry factors, such as heparan sulfate proteoglycans	Viraleze™ (Starpharma Pty Ltd., Australia)	[266,267]
*Chitosan* and *Chitosan* derivatives	Nasal spray	In vitro In vivo preclinical	Broad -spectrum antiviral activity, including against MERS and SARS-CoV-2	Binding to and blocking of the SARS-CoV-2 S-protein and RBD and subsequent inhibition of the receptor–adhesin connection and viral entry	n/a	[146,147,268,269]
	Semifacial respirator with chitosan nanoparticles	Clinical trial			n/a	
Iota-, Lambda-, and Kapa *Carrageenan*	Oral spray	In vitro Clinical trial	Broad-spectrum antiviral activity, including against SARS-CoV-2	Formation of a negatively charged protective barrier on mucus membranes, viral binding and subsequent viral aggregation, and decreased viral attachment and entry	Coldamaris^®^ (Sigmapharm Arzneimittel GmbH, Austria) Throat Spray, GripVis, Viruseptin, Iovir^®^ (Cube Pharma & Nutrition, Greece) Throat spray	[15,132,149,150,270,271,272,273,274,275,276,277,278]
	Lozenges	In vitro Clinical trial			Coldamaris^®^ (Sigmapharm Arzneimittel GmbH, Austria) lozenges, Betadine^®^ (Avrio Health L.P. Purdue Pharma Inc., USA) lozenges, Lontax Gola, Viruseptin^®^ (Beampoint AB Joint-stock company, Sweden) lozenges	
	Nasal spray	In vitro Clinical trial			Flo™ Travel nasal spray (ENT Technologies Pty Ltd., Australia), Coldamaris pro. ^®^ (Sigmapharm Arzneimittel GmbH, Austria) nasal spray, GripVis, Betadine^®^ (Avrio Health L.P. Pur-due Pharma Inc., USA) Cold Defence nasal spray, Viruseptin^®^ (Beampoint AB Joint-stock company, Sweden), Agovirax^®^ (GryNumber Health, Ltd., Lithuania), Iovir^®^ (Cube Pharma & Nutrition, Greece) nasal spray, Nasitrol nasal spray	
**IV. NON-POLYMERIC NATURAL ANTIMICROBIAL AGENTS**						
*Lactoferrin*	Intranasal liposomal suspension	Clinical trial	Broad-spectrum antimicrobial activity, including against SARS-CoV-2	Binding with endogenous cell entry factors, such as heparan sulfate proteoglycans, and SARS-CoV-2 S-protein	n/a	[279,280]
*Nitric oxide* (NO)	Nasal spray	In vitro Clinical trial	Component of the innate immunity	Mediator of immune-inflammatory cascade defense mechanisms	*Nitric oxide*-releasing systems (NORS™)—Enovid™/VirX™ (SaNOtize Research and Development Corp., Canada)	[281,282]
Type I *Interferon*	Nasal drops	Clinical trial	Component of the first-line nonspecific (innate) immunity against viruses	Prevents a delay in INF-I natural response	n/a	[283,284,285]
*Xylitol*	Nasal spray	In vitro Clinical trials	Antimicrobial activity, including against RSV and SARS-CoV-2	Anti-adhesive effects, prebiotic activity	Xlear^®^ (SaNOtize Research and Development Corp., Canada) sinus care products	[278,286,287,288]
*Trypsin* (from cod) + *Glycerol*	Oral spray	In vitro Clinical trial (on athletes)	Antiviral activity, including against HCoV-229E and SARS-CoV-2	Formation of a physical barrier on the mucosa that entraps viral particles (*Glycerol*); proteolytic activity of *Trypsin* on trypsin-susceptible sites along the SARS-CoV-2 S-protein	ColdZyme^®^ (SaNOtize Research and Development Corp., Canada) mouth spray, Viruprotect mouth spray	[289,290,291]
*Lysozyme* (as pretreatment agent prior to nasal vaccination)	Intranasal	In vivo preclinical	*Influenza*, SARS-CoV-2	Disruption of nasal bacteria and subsequent release of pathogen-associated molecular patterns, which may act as adjuvants to enhance the virus-specific antibody response to vaccination	n/a	[292]
*Hydroxytyrosol* + α-cyclodextrin	Nasal spray	In vitro	Broad-spectrum antiviral activity	Depletion of sphingolipids from the lipid rafts where the ACE2 receptor, specific for SARS-CoV-2, localizes (α-cyclodextrin)	Endovir Stop	[293]
*Longan* extract (rich in polyphenols)	Nasal spray	Clinical trial	Potential antiviral, including anti-SARS-CoV-2 activity	Potentially owed to the polyphenols’ unspecific antiviral and anti-inflammatory properties	n/a	[294]
	Oral spray				P80 Throat spray	
Flavonoid complex (patent combination Flavabac)	Oral spray	In vivo preclinical	Broad-spectrum antimicrobial activity, including against SARS-CoV-2	Antiviral activity of flavonoids is owed to their anti-oxidant properties and ability to interact with key enzymes, receptors, and membranes	Cold & Flu Guard oral spray	[295,296,297]
**V. IMMUNE-MODULATORS/ THERAPEUTICS WITH IMMUNE-MODULATORY ACTIVITY**						
INNA-051 (synthetic PEGylated TLR2/6 agonist)	Nasal spray	In vivo preclinical Clinical trial	SARS-CoV-2	Reduction of the time required for nasal epithelial cells to initiate the innate immune responses following virus exposure	n/a	[298,299,300]
*Ivermectin*	Oral (buccal) drops	Clinical trial	Antiviral activity, including against SARS-CoV-2	Interaction with SARS-CoV-2 S-protein and host cells entry factors, and subsequent inhibition of viral cell entry; blocking of the nuclear transport of the SARS-CoV-2 viral proteins by action on *Importin* superfamily; blocking of viral replication and assembly; immune-modulatory properties	n/a	[272,275,301]
**VI. OTHER**						
Probiotic strain *Lacticaseibacillus casei* AMBR2	Nasal spray	In vitro	*Staphylococcus aureus*, *Moraxella catarrhalis,* and *Haemophilus influenzae*	Secretion of antimicrobial substances such as lactic acid, bacteriocins, and H_2_O_2_	n/a LiviaOne Probiotics Nasal Spray ^b^	[183,184,302]
*Ethyl lauroyl arginate hydrochloride* (ELAH)	Nasal spray	In vivo preclinical Clinical trial	Broad-spectrum antimicrobial activity, including against SARS-CoV-2	Formation of positively charged physical barrier on mucosa and inhibition of viral adhesion	Covixyl-V	[300]
Silver nanoparticles	Nasal and oral rinse	In vitro Clinical trial	Broad-spectrum antimicrobial activity, including against SARS-CoV-2	Interference with the structural proteins of the virus and inhibiting their ability to bind with cell receptors, or bind to genetic material of viruses and inhibiting their replication	ViruStat-RDS nasal spray (+Xylitol), Silvacol nasal spray	[303,304]

^a^ product not available, ^b^ contains other *Lactobacilus* strains.

## Data Availability

Data sharing not applicable.
